# Malignant Fibrous Histiocytoma of the Maxillary Sinus in a Spray Painter from an Automobile Repair Shop

**DOI:** 10.1186/2052-4374-25-30

**Published:** 2013-11-01

**Authors:** Seok-Hwan Choi, Se-Yeong Kim, Man-Ki Son, Hui-Seok Yang, Sun-Woo Lee, Jung-Il Kim, Kap-Yeol Jung

**Affiliations:** 1 Department of Occupational and Environmental Medicine, College of Medicine, Dong-A University, Dongdaesin-dong 3-ga, Seo-gu, Busan, Korea; 2 Department of Occupational and Environmental Medicine, Busan Paik Hospital, Inje University, 75, Bokji-ro, Busanjin-gu, Busan, Korea

**Keywords:** Malignant fibrous histiocytoma, Spray painter, Occupational exposure, Chromium, Nickel, Formaldehyde

## Abstract

**Background:**

We report a case of a spray painter who developed malignant fibrous histiocytoma (MFH) of the maxillary sinus following long-term exposure to chromium, nickel, and formaldehyde, implying that these agents are probable causal agents of MFH.

**Case report:**

The patient developed right-sided prosopalgia that began twenty months ago. The symptom persisted despite medical treatment. After two months, he was diagnosed with MFH through imaging studies, surgery, and pathological microscopic findings at a university hospital in Seoul. His social, medical, and family history was unremarkable.

The patient had worked for about 18 years at an automobile repair shop as a spray painter. During this period, he had been exposed to various occupational agents, such as hexavalent chromium, nickel, and formaldehyde, without appropriate personal protective equipment. He painted 6 days a week and worked for about 8 hours a day.

Investigation of the patient’s work environment detected hexavalent chromium, chromate, nickel, and formaldehyde.

**Conclusions:**

The study revealed that the patient had been exposed to hexavalent chromium, formaldehyde, and nickel compounds through sanding and spray painting. The association between paranasal cancer and exposure to the aforementioned occupational human carcinogens has been established. We suggest, in this case, the possibility that the paint spraying acted as a causal agent for paranasal cancer.

## Background

Paranasal sinus cancer is a very rare cancer that develops in the maxillary, ethmoid, frontal, or sphenoid sinus. This cancer constitutes less than 3% of all head and neck cancers and less than 1% of cancer mortality in the United States. Over 60% of paranasal sinus cancer occurs in the maxillary sinus. The peak age of onset is 55–65 years in men and 60–80 years in women, with a median age at diagnosis of 62 years for men and 72 years for women. In people over the age of 35 years, it is 1.5–3 times more frequent in men than in women. Histologically, paranasal sinus cancer exhibits a variety of patterns: the majority is squamous cell carcinoma, but anaplastic carcinoma, transitional cell carcinoma, adenocarcinoma, adenoid cystic carcinoma, sarcoma, lymphoma, and malignant melanoma also occur. Sarcoma comprises approximately 6% of cases [1].

Among sarcomas, malignant fibrous histiocytoma (MFH) is the most common soft tissue sarcoma in adults, being more common in Caucasians than people of African or Asian descent, and occurs approximately 2 times more frequently in men than in women. Clinically, it occurs commonly in the femoral or hip region in patients aged 50–70 years, but can also occur in the head, neck, or face, albeit rarely [2]. Of the paranasal sinuses, MFH occurs frequently in the maxillary sinus and constitutes 0.27% of malignant head and neck cancers [3]. MFH of the maxillary sinus is often a firm and asymptomatic nodal mass and accompanies clinical symptoms similar to other paranasal sinus cancers. Histologically, it is classified into 4 types: storiform-pleomorphic, myxoid, giant cell, and inflammatory. The storiform-pleomorphic and myxoid types constitute 70% and 20% of MFH, respectively, and the giant cell and inflammatory types are rare [2]. The current hypotheses regarding its carcinogenesis include the histiocytic origin hypothesis by Kauffman and Stout, which states that the cancer originates from tissue histiocytes, the undifferentiated mesenchymal cell origin hypothesis by Fu, and the fibroblast origin hypothesis by Hoffman and Dickersin. The fibroblast origin hypothesis or the primitive mesenchymal cell origin hypothesis is favored [4-6].

Paranasal sinus cancer is a rare cancer in the general population. The prevalence rate is higher in males, who tend to work outside the home more frequently, than in females, and case reports for specific work groups are more frequent. Therefore the relative risk of those exposed to the occupationally hazardous substances described has been as high as 30–1000 times that of the general population. Nickel refining, exposure to nickel and nickel compounds, mustard gas manufacturing, isopropyl alcohol production, watch face painting, and thorium dioxide contrast medium have been demonstrated to be factors associated with squamous cell carcinoma. Exposure to hardwood dust, leather dust, flour milling, polycyclic aromatic hydrocarbons (PAH), and asbestos are known to be associated with adenocarcinoma [1]. Of these factors, wood dust and leather dust are thought to be closely related factors, and other factors, such as nickel and nickel compounds, hexavalent chromium and formaldehyde, welding fumes, oil mists, flour, cocoa powder, textile dust, coal dust, paint mists, strong acids, mustard gas, isopropyl alcohol, and thorium dioxide contrast agents have been suggested to be risk factors [7]. Factors that affect the development of sarcoma in adults include genetic disorders such as neurofibromatosis type 1 (von Recklinghausen’s disease), Li-Fraumeni syndrome, and retinoblastoma, as well as infections or a history of radiation exposure. In a study of occupational factors, there was a slight, but statistically insignificant, increase in the risk of sarcoma in those who were farmers, gardeners, railroad workers, construction workers, or who had been exposed to herbicides and chlorophenol, but different studies have reported incongruent results. Hence, no clear association with occupational causes has been established [8].

Except for wood dust and leather dust, no clear association has been observed for the abovementioned occupational risk factors of paranasal sinus cancer. In contrast to cancers of other histological types such as squamous cell carcinoma or adenocarcinoma of the same site, no occupational or environmental medicine case reports or studies have been published regarding paranasal sinus sarcoma, particularly MFH. We encountered a case of MFH of the maxillary sinus in a man who had worked for 18 years and 6 months as a spray painter in an automobile repair shop. We report the first known case of its kind worldwide, and discuss its association with exposure to hazardous substances during the painting process.

## Case presentation

### Patient

Male, 41 years old (at the time of diagnosis).

### Chief complaint

Right-sided prosopalgia.

### History of present illness

The chief complaint of the patient was right-sided prosopalgia, which had developed twenty months ago, and the patient had been treated with medication for sinusitis at an ear, nose, and throat clinic several times without any improvement. Hence, two months after the initial symptoms, the patient was admitted to a university hospital in Pusan, Korea, and underwent computed tomography (CT) and magnetic resonance imaging (MRI) examinations, endoscopic middle meatal antrostomy, and frozen tissue biopsy, and was diagnosed with a mesenchymal tumor of the right maxillary sinus. Following diagnosis, the patient was hospitalized at the otolaryngology department of a university hospital in Seoul, Korea, for treatment. The patient underwent CT, MRI, positron emission tomography CT (PET-CT), angiography, and various blood tests. The tumor was removed by total maxillectomy and neck dissection. Pathological examination of the tumor confirmed the diagnosis of high-grade myxoid-type MFH. Currently, the patient is undergoing postsurgical anticancer chemotherapy and radiation therapy.

### Past history and family history

The patient did not have a prior history of remarkable medical conditions or surgeries.

### Routine medical examination records

There were no specific findings from the patient’s workplace routine medical examination and cancer examinations.

### Social history

The patient had smoked 1–3 cigarettes a day for less than 5 years. The patient stated that he rarely drank.

### Physical examination

The vital signs in the medical records were normal. There were no specific findings during the physical examination.

### Laboratory medical examination

There were no specific findings from the blood tests, biochemical tests, or urinalysis. No tests for genetic disorders were conducted.

### Radiological examination

**Figure 1:**
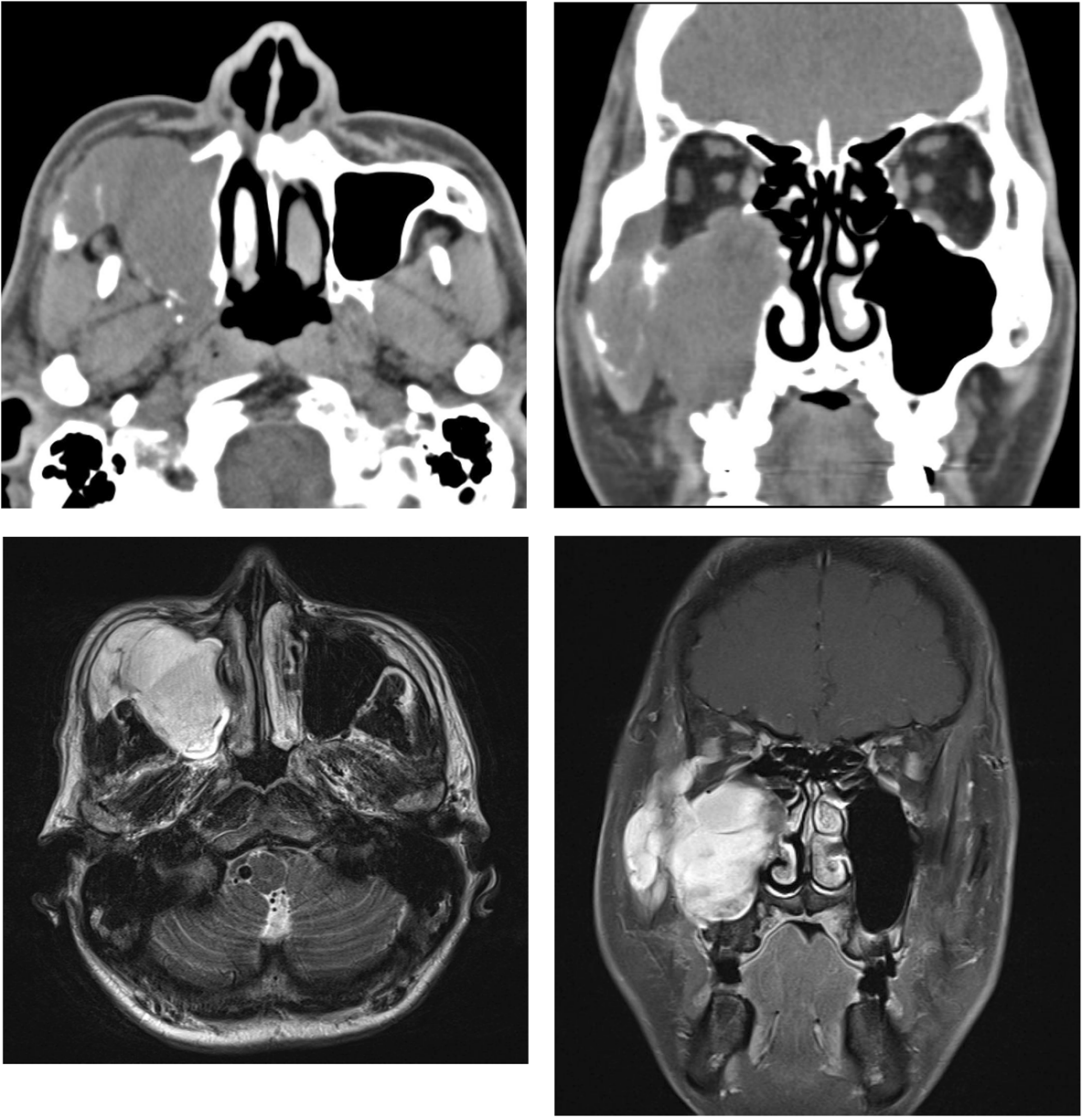
**Paranasal CT and MRI findings of maxillary sinus MFH.** The figures show a large mass involving the anterior, posterolateral, and medial walls of the right maxillary sinus; inferior and lateral walls of the right orbit; anterior aspect of the right zygomatic arch; right pterygoid bone; and anterior surface of the right sphenoid bone.

CT, MRI, and 3D-CT examinations of the head and neck regions conducted in April revealed a mass that had invaded the medial, anterior, and posterior walls of the right axillary sinus, reaching the lateral wall of the orbit and the orbital floor, and whose apex was at the anterior aspect of the zygomatic arch up to the anterior surface of the sphenoid and pterygoid bones. No metastasis to the brain was observed (Figure 1). Ultrasound, endoscopy, angiography, and PET-CT conducted to assess distant metastasis revealed no metastasis.

### Pathological examination

Histological evaluation of the tumor confirmed the diagnosis of high-grade myxoid-type MFH.

### Occupational history and type of work

**Figure 2:**
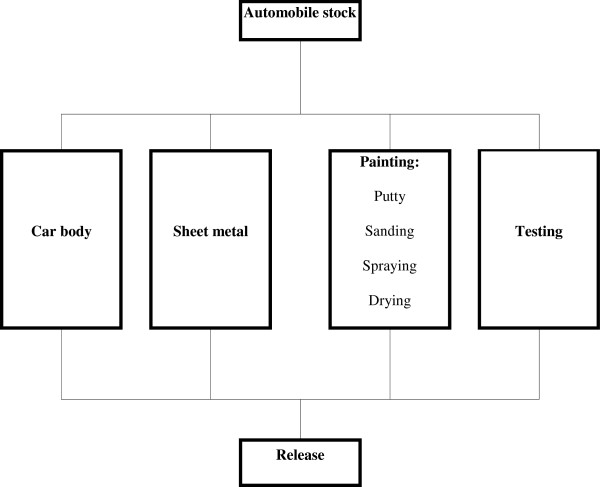
Automobile repair process.

The patient worked at K. Automobile Repair Shop in Gyeongnam, Korea (Workplace 1) beginning 1993. From 2008 until the cancer diagnosis, the patient worked at I. Automobile Service in Busan, Korea (Workplace 2), working solely in the painting department of each workplace for approximately 18 years and 6 months in total. Both workplaces were auto body repair service shops, and depending on the site and extent of damage to the automobile, underbody repair, plating, and painting were carried out separately. One worker was designated for each process and worked in a booth designated for the process (Figure 2). The painting process involves surface preparation, painting and drying, which are carried out either together or separately depending on the vehicle model and the extent of damage. Preparation includes organizing and cleaning the damaged site, filling the damage and dents with putty, sanding to smooth the surface, and if necessary, drying using a small drying lamp. Painting is done using the 3-coat method, with bottom, middle, and top coats applied in the painting booth, and drying is carried out using a dryer installed in the booth. At both workplaces, no worker solely worked on one part of the painting process. Typically, the preparation, painting, and drying of one car was carried out by one person.

Workdays lasted 7 hours and 30 minutes per day on Mondays through Fridays, excluding holidays, spanning 8:30 AM to 5:00 PM, excluding a 1-hour lunch time. On Saturdays, workers typically worked for 3 hours and 30 minutes between 8:30 AM and 12:00 PM. Excess administrative work was performed on average for 9 hours a week, 40 hours a month. Breaks other than lunch time were taken irregularly.

**Figure 3:**
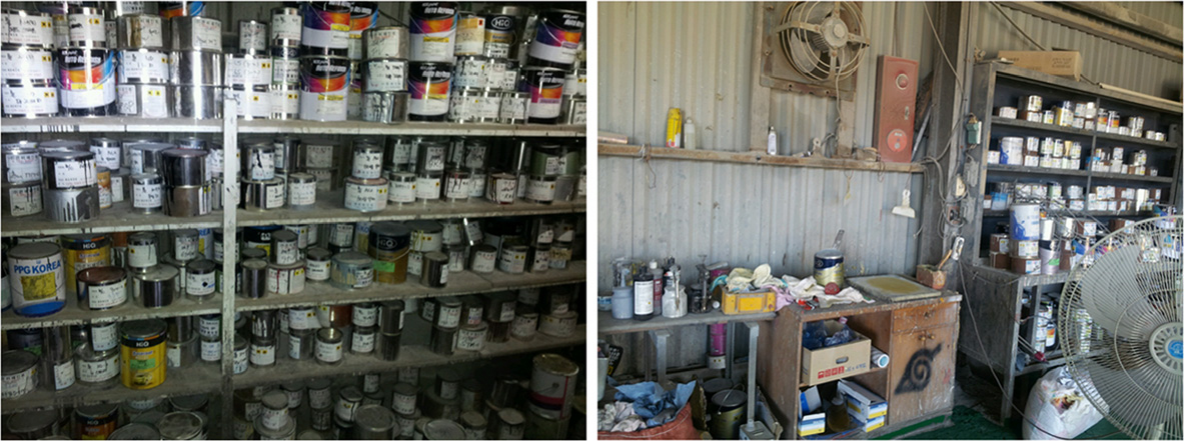
Paint worktable and inventory.

**Figure 4:**
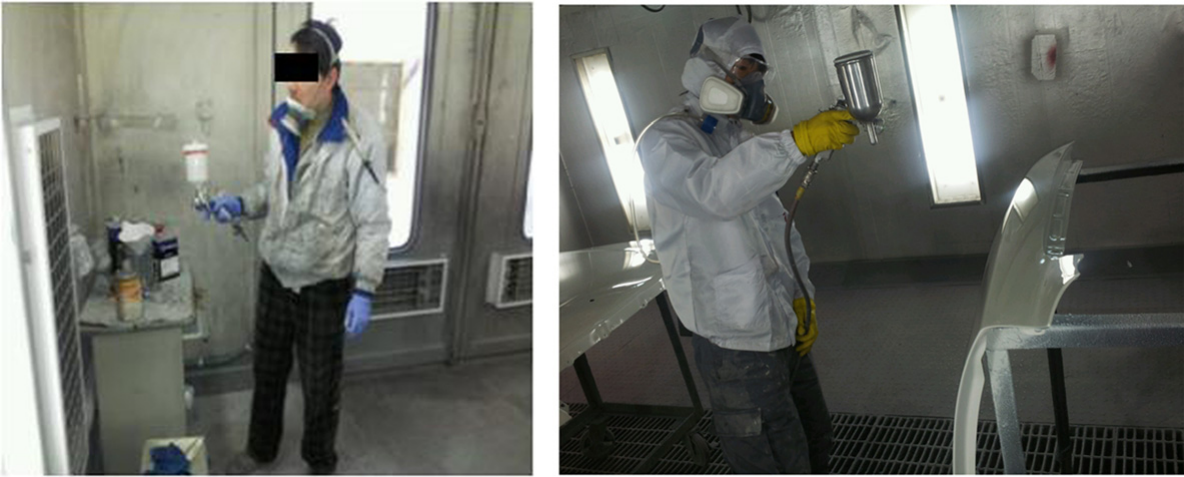
Spray painting process.

**Figure 5:**
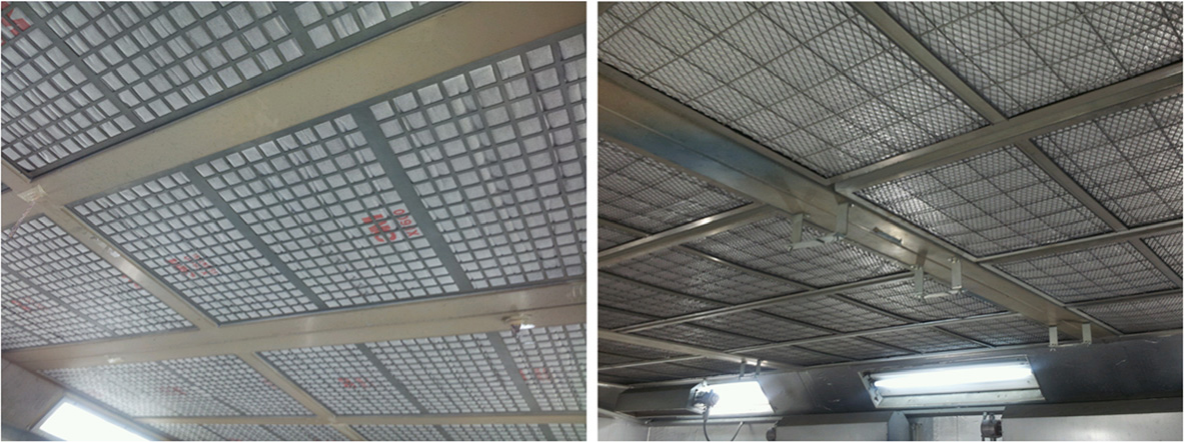
Ceiling inlet in the painting booth.

**Figure 6:**
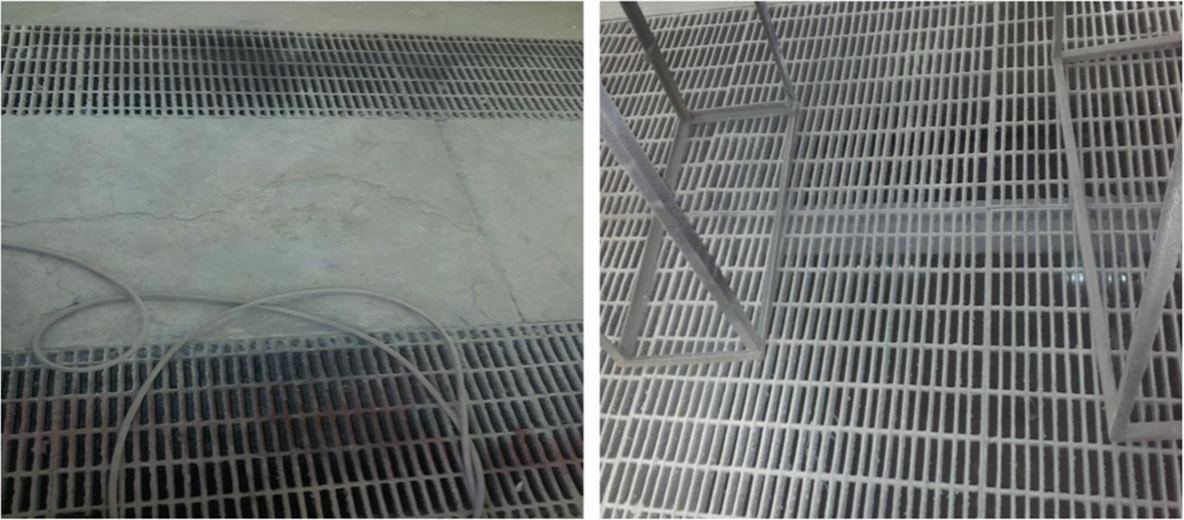
Floor exhaust in the painting booth.

**Figure 7:**
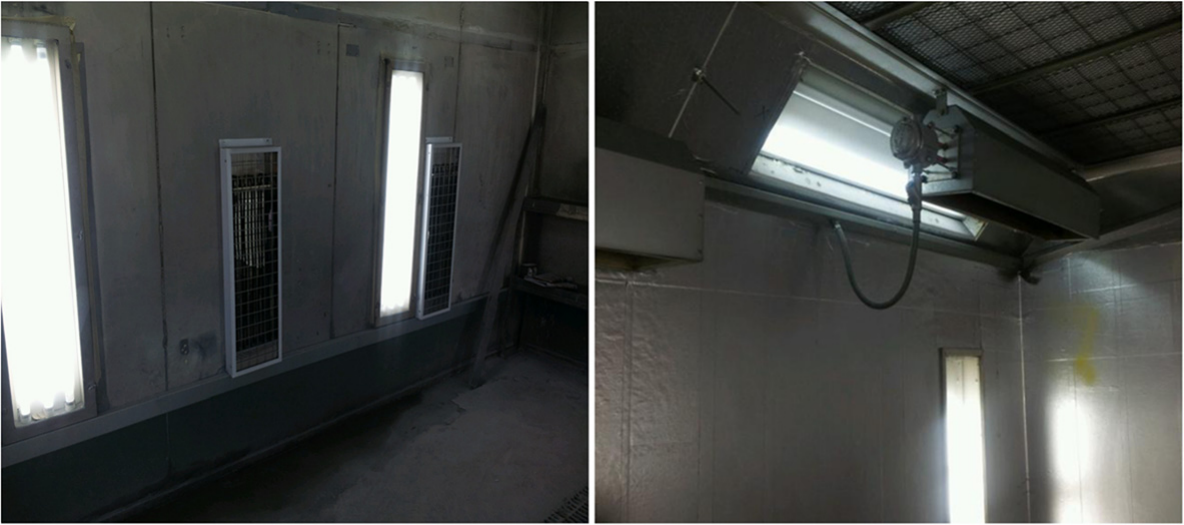
Dryer in the painting booth.

**Figure 8:**
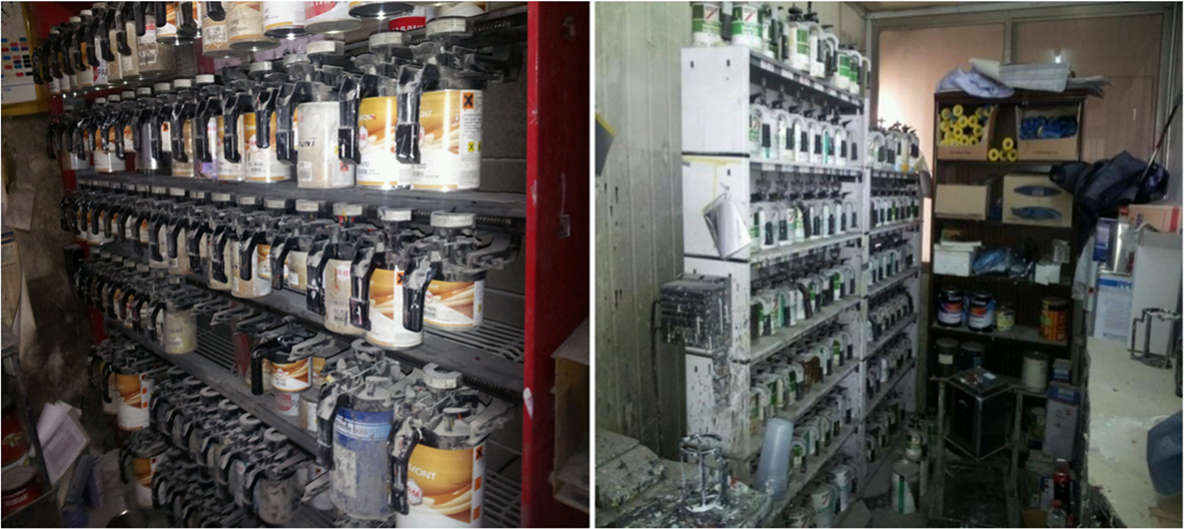
Toner room.

**Figure 9:**
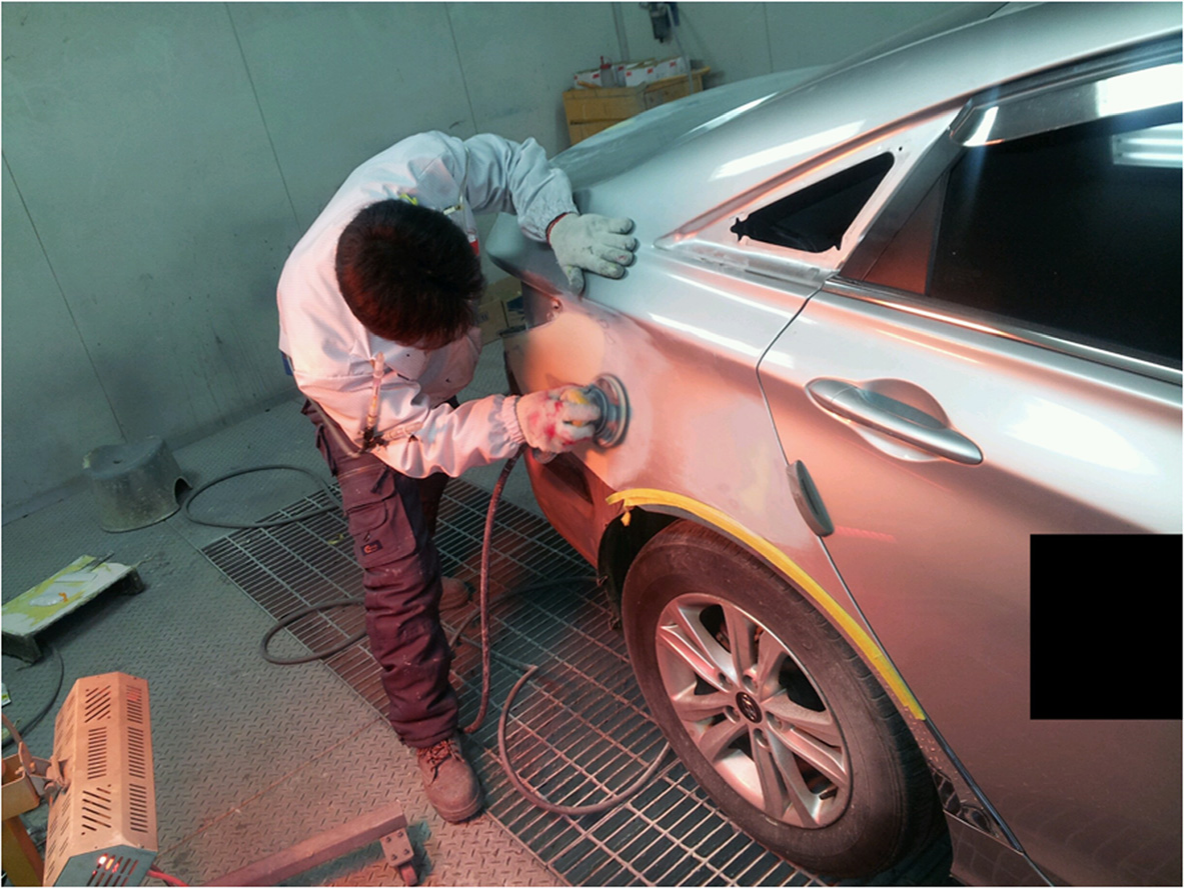
Sanding process.

There are 4 steps to the automobile painting process, beginning with preparation, which includes organizing the damaged surface, cleaning, treatment with putty, and sanding; selection of paint color and toning; spray painting; and drying. Spray painting and drying are done inside the booth, and the other processes are done outside the booth. Painters first repair the structural damage to the area to be painted, apply putty to the surface, and when it is dry, grind the surface smooth using a grinder. Then, the appropriate paint color is selected from the paint worktable and inventory (Figure 3). After the preparation, spray painting is usually done in the booth (Figure 4). The booths are designed with a push–pull structure, where air enters from the ceiling (Figure 5) and is drawn down by exhaust fans in the floor (Figure 6). The spray pressure is 2.5 bar during spray painting. The drying process varies with the season, but generally takes place at 65–70°C (Figure 7). Drying is done in the booth. During this process, workers frequently enter the booth without gas mask in the shop where the patient worked. For luxury cars, which constitute 5% of the automobiles serviced, the paint is mixed in the paint toning room to select a paint color suitable for the model (Figure 8). The occupational environment is poor. Aside from the lunch time, there are no other specified breaks. With the exception of painting booth work, workers frequently work without proper protective equipment, such as goggles for eye protection, gas and filter respirators, protective gloves, and a protective suit. Disposable dust masks are used during some of the sanding processes, and dust collectors, which should normally be used with the sander, are frequently not connected (Figure 9).

The 2 workplaces used HI-Q Primer-Surfacer Beige paint, HI-Q putty, HI-Q automobile-grade urethane thinner (Noroo Paint Inc.), KAR Crystal Vanilla White paint, KAR Primer-Surfacer Beige paint, Eco Putty, acrethane thinner (Jebi Paint), Auto Colorbase paint, Super putty, urethane thinner (KCC), Low-VOC RAD paint, Royal Thane Q putty, and Royal Thane thinner (PPG Korea). To determine the occupational exposure, the amounts of paint, thinner, and solvent were estimated on the basis of the transaction records of each workplace. The occupational exposure to the spray painting process was calculated according to the monthly paint and solvent use. The occupational exposure of the preparation process was calculated according to the monthly putty use. On average, Workplace 1 used 80 L paint, 40 L solvent, and 10 L putty per month; Workplace 2 used 240 L paint, 144 L solvent, and 48 L putty per month. These amounts were similar to or less than the average monthly usage of less than 250 L paint in typical auto body service shops in Korea.

### Working environment measurement and analysis

We reviewed the workplace records of the working environment measurements from the past 9 years. Toluene-2,4-diisocyanate, talc, toluene 2,6-diisocyanate, methylene bisphenyl diisocyanate, and hexamethylene diisocyanate were detected at levels lower than the exposure limits. However, measurements related to carcinogens causing paranasal sinus cancer, such as those for hexavalent chromium, nickel compounds, and formaldehyde, had not been conducted. Therefore, to evaluate the working environment and exposure of the patient to carcinogens, we visited the 2 workplaces on January 2013. We interviewed the other workers and measured the exposure levels during the painting process, including inside the booth. The transaction records and material safety data sheets of the paint, organic solvent, and putty showed that chromium-containing paint was present (Table 1).

**Table 1:** Chromium component and content of paints used in automobile repair shops

**Products (Code No.)**	**Manufacturer**	**Chromium component**	**Content (%)**
Autocryl MM (00388/000573)	Akzo Nobel Coatings b.v.	C.I.Pigment Yellow 34	25-50
Washprimer CR (01907/000000)	Akzo Nobel Coatings b.v.	Zinc(potassium) chromate (49663-84-5)	2.5-10
Carmix 993 Tinter Master: YVV942 YVV944	Mercury paints factory SDN BHD	Lead Chromate Pigment (7758-97-6)	30-50
PC Brilliant Mix MB710 Orange	Spies Hecker Gmbh	Chromium complex compound (110342-29-5)	5-7
Permacron brilliant MB 710	Performance Coatings Gmbh & Co. KG	Chromium complex compound (110342-29-5)	5-7

The workers and owners stated that the current processes and equipment were identical to when the patient had worked there previously. According to the patient’s colleagues at Workplace 1, respirators or other protective equipment other than a dust mask had not been provided since 1993. Beginning in 2000, the use of respirators was recommended. The colleagues also stated that the volume of work on the day of measurement was similar to that of a typical day. We obtained 12 samples for 8-h continuous measurement of substances from 4 workers of 2 workplaces, and 6 workplace air samples: a total of 18 samples. The collected samples were tightly sealed with the cap in the sampler and were transported under ambient pressure and temperature. The measurement and analysis were conducted according to the methods of Notification 2011–55 from the Korean Ministry of Labor and the United States National Institute for Occupational Safety and Health (NIOSH) standard.

Hexavalent chromium was measured according to NIOSH method 7605 using a personal air sampler (GilAir-3 RC; Gilian Instrument Corporation, West Caldwell, NJ, USA) with a polyvinyl chloride filter (5.0 μm × 37 mm, SKC Lot No. T20810; SKC Inc., Eighty Four, PA, USA). The measurements were read using atomic absorption spectroscopy (Shimadzu AA-7000 F; Shimadzu, Japan) at 540 nm. Nickel compound was measured according to NIOSH method 7300 using a personal air sampler (GilAir-3 RC) with a mixed cellulose ester filter (Low BGD, 0.8 μm × 37 mm, SKC Lot No. 12722-7 DC-223; SKC Inc.). The measurements were read using atomic absorption spectroscopy (Shimadzu AA-7000 F; Shimadzu) at 231 nm. Formaldehyde was measured according to NIOSH method 2016 using a personal air sampler (GilAir-3 RC) with a 2,4-dinitrophenylhydrazine―silica gel tube (SKC Lot No. 7687, Cat. No. 226–119; SKC Inc.). The measurements were read using a high-performance liquid chromatography–ultraviolet absorbance detector (1260 Infinity; Agilent Technology, USA) at 360 nm.

We detected insoluble hexavalent chromium, nickel (insoluble inorganic compounds), and formaldehyde at both workplaces. The maximum level of each hazardous compound detected was as follows: during the painting process at Workplace 1: 0.0013 mg/m^3^ hexavalent chromium, 0.0047 mg/m^3^ nickel (insoluble inorganic compounds), and 0.3244 ppm formaldehyde; during the painting process at Workplace 2: 0.0006 mg/m^3^ lead chromate, 0.0001 mg/m^3^ nickel (insoluble inorganic compounds), and 0.1069 ppm formaldehyde. All measures were lower than the exposure limits set by the Korean Ministry of Employment and Labor (Table 2).

**Table 2:** Working environment measurement for each automobile repair shop

	Workplace 1^*^	**Workplace 2**^**†**^	**Normal limit**
2,4-TDI^‡^	-	-	
2,6-TDI	-	-	
MDI^§^	-	-	
HDI^**^	-	-	
Talc (except asbestos)	-	-	
chromium	-	-	
Trivalent chromium	-	-	
Chromate (hexavalent chromium) (mg/m^3^)	0.0008-0.0013	0.0003-0.0006	0.0500
Lead (inorganic dusts, fume)	-	-	
Nickel (insoluble inorganic compound) (mg/m^3^)	0.0039-0.0047	0.0001	0.5000
Formaldehyde (ppm)	0.3016-0.3244	0.0965-0.1069	0.7500

*K. automotive repair shop.

†I. automotive repair shop.

‡Toluene d-iso cyanate.

§4,4′-Methylene di(bis)phenyl diisocyanate.

**Hexamethylene diisocyanate.

During the spray painting in a booth, the ventilated air, that is, airflow control velocity, affects the painters’ exposure to hazardous substances [9]. Therefore, we used an anemometer (velocity CALC plus, HH-616-M; TSI, USA), which analyzes the airflow, placing it in the painting booths of the 2 workplaces to measure the airflow control velocity inside while spray painting and drying were carried out. Five measurements were obtained from the ceiling inlet, floor exhaust, and the middle region, and the geometric average was calculated. During spray painting, the velocity control of airflow from the upper to lower region differed between the outer edges and the middle of the booth. Compared to the standards in the Occupational Safety and Health Standards Regulation of the Ministry of Employment and Labor, where the standard velocity control for local enclosing-type exhaust hood systems for hazardous substances is 0.4 m/s for gaseous substances and 0.7 m/s for particulate substances [9], the velocity control was too low. In particular, it was lowest in the middle region, where a painter normally does spraying work (Table 3).

**Table 3:** Velocity control of local exhaust in painting booths unit: m/s

**Process**	**Velocity control**			**Normal limit**
	**Ceiling inlet**	**Midpoint**	**Floor exhaust**	
Spray painting^*^	0.1-0.5	0.1-0.2	0.1-0.3	0.4^†^
Drying^*^	0	0	0	0.7 ^‡^
Spray painting^§^	0.3-0.5	0.1-0.3	0.1-0.4	
Drying^§^	0	0	0	

* Velocity control of local exhaust in K. automotive repair shop.

†Velocity control of gaseous matter.

‡Velocity control of particulate matter.

§Velocity control of local exhaust in I. automotive repair shop.

### Progress and treatment

The patient filed for industrial accident claims at the Korean Workers’ Compensation and Welfare Service (KComWel) for MFH of the maxillary sinus, and KComWel requested that the Occupational Safety and Health Research Institute carry out an epidemiological survey to assess whether the disease could be considered an industrial accident. The Department of Occupational and Environmental Medicine of a university in Busan conducted the epidemiological survey. It was determined that the condition was related to the occupation, and the claim was approved.

## Conclusion

There have been no case reports of paranasal sinus cancer and MFH in paint workers in the automobile repair industry. This case was one of maxillary sinus cancer diagnosed in a 41-year-old man, younger than the typical male patient age of 55–65 years. Furthermore, the patient did not smoke very much, smoking approximately 1–3 cigarettes per day for less than 5 years. Smoking has been thought to be a risk factor for paranasal sinus cancer. The patient also neither suffered from chronic sinusitis nor had a history of radiation exposure.

Of the factors related to paranasal sinus cancer carcinogenesis, chromium and chromate are often present in the paint used by automobile repair [10]. The dust that is produced during preparation when sandblasting the filler, the painted automobile body, and metal surfaces contains chromium, hexavalent chromium, and nickel [11]. During the heating of putty and drying, formaldehyde is generated from the resin in the paint [12]. Therefore, the painter is constantly exposed to nickel compounds, hexavalent chromium, and formaldehyde during the spray painting process. Nickel compounds, hexavalent chromium, and formaldehyde are all considered Group 1 carcinogens (clearly carcinogenic in humans) according to the International Agency for Research on Cancer, and for paranasal sinus cancer, nickel compounds are classified to have sufficient evidence, and hexavalent chromium and formaldehyde are classified to have limited evidence.

Paint is a fluid chemical product, used to create a surface film on an object to maintain gloss, provide insulation from heat and electrical radiation, and prevent mold growth. Paints are typically composed of film-forming materials or pigment vehicles such as oil or resin, and pigments such as coloring, inhibitive, or heat-resistant pigments, or extenders, and additive, and solvent. The film-forming materials commonly used in Korea include synthetic resins such as epoxy, alkyd, acrylic, polyamide, vinyl chloride, and polyurethane resins, and the polyurethane family of resins has been used more frequently recently. Pigments are insoluble fine-powder solids that are dispersed into paint, which contribute to color, durability, and decay resistance, and play a role as filler or extender pigments. In Korea, heavy metals are typically used in pigments. Paints for automobile electrophoretic painting or repair painting contain lead, zinc, manganese, nickel, tin, or copper. Pigment comprises 20–60% of the paint, and when applied, rarely poses a hazard; however, the particles are aerosolized during painting, and can be inhaled into the respiratory system. Polyurethane paint contains a higher percentage of free hexavalent chromium than other paints [10,13,14]. In the Korean literature, a study of the working environment of spray painters in shipping container manufacturing workplaces reported a hexavalent chromium level of 0.264–0.318 mg/m^3^ in the air [15].

Automobile repair industry workers use a 3-step process for car body surface structural repair: preparation which involves filling, sanding, and cleaning with organic solvents; spray painting; and drying. Some of the processes include treatments corrosive to the auto body [16]. In an analysis of the occupational hygiene of automobile repair shop workers in the United States, heavy metals such as chromium, hexavalent chromium, and nickel were detected in the dust produced during the surface sanding process, and examination of work clothes revealed heavy metals such as chromium and nickel [11].

A low level of chromium is an essential mineral for the human body, but it has been reported that high-dose exposure to hexavalent chromium can lead to respiratory or paranasal sinus cancers. Hexavalent chromium has been declared a confirmed carcinogen (Group 1) for humans; in animal experiments, calcium chromate, lead chromate, strontium chromate, and zinc chromate have been classified as having sufficient evidence for carcinogenesis, and anhydrous chromium and sodium dichromate were classified as having limited evidence for carcinogenesis. The carcinogenic mechanism of chromium has not been fully described, but several studies have suggested that DNA modification or damage is involved. One study reported that microsatellite instability due to DNA mismatch repair system alterations was higher in the chromium-exposed group than in the non-exposed group [17-21]. d’Errico *et al.* investigated the risk of various occupational hazards on nasal cavity or paranasal sinus cancer by histological type in 113 workers who developed nasal cavity or paranasal cancer and 336 hospital controls. In undifferentiated, mucoepidermoid, neuroendocrine, basocellular, and unspecified tumors, excluding adenocarcinoma and squamous cell carcinoma, chromium exposure and organic solvent exposure led to a 9.2-fold and 5.7-fold increase in carcinogenesis, respectively [22]. It has been reported that the prevalence of paranasal sinus cancer is higher in workers working with chromium compound manufacture, chromium-containing paint products, and chromium plating. It has been suggested that the probability of carcinogenesis of paranasal sinus cancer is often associated with nasal cavity mucosal cell abnormalities [23,24].

Formaldehyde is a secondary substance created during the automobile painting process [12]; it can also arise from daily activities such as exposure to cigarette smoke, paint, garments, medications, and automobile exhaust. In animal and cell culture experiments, exposure to formaldehyde leads to transformation and damage in several steps of expression of the genes involved in xenobiotic metabolism, cell cycle regulation, DNA synthesis and repair, oncogenes, and apoptosis. In mammalian nasal cavity tissue, formaldehyde exposure has been observed to cause an increase in DNA adducts, DNA–protein cross-links, DNA–DNA cross-links, and DNA single-strand breaks [25-27]. In the nasal cavities of primates, formaldehyde exposure has been shown to lead to microRNA changes related to the apoptosis of nasal cavity cells [28]. Formaldehyde increases the risk of adenocarcinoma in the paranasal sinus and is reported to be associated with squamous cell carcinoma, although the mechanism is unclear [29]. Formaldehyde has been frequently associated with accessory carcinogens, and it has been reported that it cannot be ruled out as a sole carcinogen [30].

Nickel is often used in daily consumer products and as raw manufacturing material in metal or compound form. The carcinogenicity of nickel in relation to paranasal sinus cancer and respiratory system cancers has been reported. Epidemiological and animal experiments have confirmed its dose-dependence and carcinogenicity [31-33]. The currently known carcinogenic mechanisms of nickel include the generation of reactive oxygen species and oxidative stress leading to DNA damage, genotoxic effects leading to chromosome abnormalities, changes in transcription factors and signaling pathways involved in gene expression, and DNA repair inhibition [34-36]. Most reports of the association between nickel compounds and paranasal sinus cancer have centered on nickel refinement workers, and cases also include workers who manufacture metals for dining tables and batteries, and workers who do nickel plating [23,24].

DNA and chromosomal changes or damage explain the expression of various cancers in various organs, and a study has suggested that structural and numerical chromosomal aberrations were much more frequent in spray painters than in control groups [37].

The working environment measurements of the 2 workplaces showed that hexavalent chromium, nickel compounds, and formaldehyde, known to be carcinogenic in paranasal sinus cancer, had not been measured. Therefore, we additionally measured the hexavalent chromium, nickel compounds, and formaldehyde of these workplaces. We found that all 3 compounds were present in the air in both workplaces, albeit at lower levels than the exposure limits. Those exposed to nickel compounds have a higher rate of squamous cell carcinoma. For hexavalent chromium and formaldehyde, the histological type of the most frequently occurring cancers differs according to the study, but squamous cell carcinoma and adenocarcinoma are prevalent. Although the histological type of frequently occurring cancer differs, it has been suggested that heavy metals such as chromium and nickel are carcinogenic, and it has also been hypothesized that they can act as co-carcinogens with organic compounds such as PAH [38]. In subsequent studies, chromium has been reported to inhibit expression of the cytochrome P450 (CYP1a1) gene and promote the carcinogenic effect of PAH [39]. Nickel affects intracellular post-translational modification, and it has been demonstrated that arsenic compounds are also involved in the same process [40]. Considering these facts, the 3 compounds detected may be synergistic in their hazardous effects in respiratory dysfunction, including tumors of the lung or nasal cavity.

Furthermore, the airflow control velocity of the 2 workplaces visited was lower than that of regulatory standards, indicating that due to the low airflow control velocity, the vapor or mist generated during automobile body work and spray painting could be expected to be present at high concentrations inside the booth. Furthermore, during drying in the booth, the solvent is dried with the local exhaust system switched off. Therefore, workers who intermittently enter the booth during the drying process are probably also exposed to secondary hazardous chemicals.

Urethane paint is frequently used in automobile service shops due to its low temperature thermosetting, low viscosity, high degree of freedom in dry paint film design, high durability, ease of painting, high efficiency, and ease of management. However, compared to other paints, it releases more hexavalent chromium [14]. Urethane paint was used more frequently in the past. In 2000, as the patient worked in a booth with insufficient airflow control velocity and without proper respirators, we assume that biological inhalation was high. Although the difference was not statistically significant, a study showed that the biological exposure rate determined by urinary chromium was higher in workers who did not use protective equipment than in workers who did [41]. In addition to the aforementioned occupational factors, smoking is also a factor that affects paranasal sinus cancer rates. However, even though the patient was a smoker, the tumor was diagnosed as a sarcoma following histological examination, and sarcoma has not been clearly associated with drinking or smoking, with the exception of chewing tobacco [8]. In conclusion, other than the spray painting process, there was no history of exposure to occupational hazard factors or any remarkable personal medical history in this case that would have affected the carcinogenesis. Considering that the age of onset differed from that of typical paranasal sinus cancers, smoking history was short, and given the length of occupation, number of occasions of exposure, lack of use of protective equipment, and painting booth conditions, low-level occupational exposure to hexavalent chromium, formaldehyde, and nickel compounds for an extended period may have increased the risk of maxillary sinus cancer development.

The current case report has several limitations in terms of working environment measurements. The range of paints used on the day of measurement was not very wide and only a limited number of paint colors and other painting materials had been used, so the working environment of the past had not been reproduced completely. Therefore, the findings obtained from the additional measurements were lower than expected. Furthermore, as there was a lack of availability of protective equipment in the past, we assume that there is a possibility of respiratory and skin exposure, but we were unable to assess the exposure or perform biological monitoring. As the current workers use protective suits and respirators, the exposure rates in the past cannot be reproduced completely.

This case study is the first known report of MFH of the paranasal sinus in a spray painter from an automobile repair shop. This study is significant in that we assessed the carcinogenicity of hexavalent chromium and nickel, heavy metals and formaldehyde, an organic solvent generated during preparation and spray painting in automobile repair shops. Through the occupational environmental measurements of this case, we determined that hazardous substances such as chromium, nickel, and formaldehyde were present in the paint and filler, and during sanding, painting, and heating; the compounds were generated primarily or secondarily, leading to worker exposure. Despite these exposures, the working environment measurements did not include measurements of heavy metals such as chromium or formaldehyde. In specialized medical examinations of the workers, measurements of chromium, nickel, and formaldehyde, shown as hazardous in this study, had not been conducted. Even though paranasal sinus cancer is highly associated with occupation, its low prevalence and long incubation mean that workers would have frequently changed jobs or discontinued working at the time of diagnosis. Therefore, work history follow-up is difficult and occupational cases or research are infrequent [42]. Hence, we would like to use this case report as a warning regarding the health hazards of chromium, nickel, and formaldehyde exposure in painters in automobile repair shops, and the risk of developing paranasal sinus cancer following such exposure.

## Consent

Written informed consent was obtained from the patient, co-workers for the publication of this report and any accompanying images.

## Competing interests

The authors declare that they have no competing interests.

## Authors’ contributions

SH C and SY K intervewed and wrote the article. MK S and HS Y searched and assisted the related references. SW L performed the analysis and estimation of the environmental assessment. JI K and KY J supported and advised medical view. All of the authors read and appoved the final manuscript.
